# Phasic Arterial Mechanics and Ventriculo-Arterial Dyssynchrony in Heart Failure with Preserved Ejection Fraction: A Systematic Review and Meta-Analysis of Arterial Systole, Arterial Diastole and Pharmacological Phase Restoration

**DOI:** 10.3390/diagnostics16213599

**Published:** 2026-11-01

**Authors:** Georgică Târtea, Cristina Văcărescu, Dragoș Cozma

**Affiliations:** 1 Department of Cardiology, University of Medicine and Pharmacy of Craiova, 2 Petru Rareș Street, 200349 Craiova, Romania; georgica.tartea@umfcv.ro; 2 Research Center of the Institute of Cardiovascular Diseases Timișoara, 13A Gheorghe Adam Street, 300310 Timișoara, Romania; dragos.cozma@umft.ro; 3 Cardiology Clinic of the Institute of Cardiovascular Diseases Timișoara, 300310 Timișoara, Romania

**Keywords:** heart failure with preserved ejection fraction, arterial stiffness, pulse wave velocity, augmentation index, wave reflection, ventriculo-arterial coupling, global longitudinal strain, arterial systole, arterial diastole

## Abstract

**Background/Objectives:** Arterial stiffness is usually treated as a scalar load. We instead framed it as a timing problem, defining arterial diastole as compliant reservoir filling during left ventricular (LV) systole and arterial systole as elastic recoil during LV diastole, and we asked whether the two phases were measurably disturbed in heart failure with preserved ejection fraction (HFpEF), and whether pre-specified drug classes could restore them. **Methods:** We searched MEDLINE/PubMed, Scopus, and Web of Science from inception to 10 September 2026 (3835 records). After deduplication, 2043 records were screened and 376 full texts were assessed, of which 236 met the eligibility criteria and 84 contributed quantitative data. Random-effects meta-analyses used DerSimonian–Laird estimations with Hartung–Knapp–Sidik–Jonkman confidence intervals; the risk of bias was assessed with the Newcastle–Ottawa Scale and Cochrane RoB 2. **Results:** Compared with controls without heart failure, patients with HFpEF or diastolic dysfunction with preserved ejection fraction had higher carotid–femoral pulse wave velocity (mean difference +1.72 m/s, 95% CI +0.73 to +2.70; k = 16; *p* = 0.002), higher brachial–ankle pulse wave velocity (+2.24 m/s, +1.38 to +3.10; k = 7; *p* = 0.0007), a higher augmentation index (+4.51%, +1.36 to +7.65; k = 10; *p* = 0.010), and a higher cardio-ankle vascular index (+1.00, +0.29 to +1.71; k = 5; *p* = 0.017). In randomised trials, pre-specified drug classes lowered the heart-rate-corrected augmentation index relative to comparators (−2.18%, −3.51 to −0.85; k = 11; *p* = 0.004), although this did not persist when ten further eligible trials were added by generic inverse variance (−1.36%, −2.78 to +0.07; k = 15; *p* = 0.060). They did not change carotid–femoral pulse wave velocity (−0.11 m/s, −0.40 to +0.18; k = 18; *p* = 0.428), an estimate that became significant when trials at an overall high risk of bias were excluded (−0.21 m/s, −0.41 to −0.02; k = 13; *p* = 0.036). Reflected-wave transit times did not differ significantly (−5.09 ms; k = 4), and the pooled correlation between arterial stiffness and longitudinal strain was not significant (r = −0.28, −0.65 to +0.20; k = 4; *p* = 0.162). Heterogeneity was substantial throughout, and 95% prediction intervals included the nulls for five of the six pooled indices, with the single exception being brachial–ankle pulse wave velocity. **Conclusions:** Arterial stiffness indices were on average higher in HFpEF, with substantial heterogeneity and prediction intervals that included the nulls for five of the six indices; under restrictions of clinically or haemodynamically defined HFpEF, the augmentation index differences lost significance (*p* = 0.071) while carotid–femoral pulse wave velocity was only marginal (*p* = 0.050). In randomised trials, the component that responded to current pharmacotherapy was the wave-reflection amplitude and not aortic conduit stiffness; this reduction derived entirely from hypertensive populations and did not survive the inclusion of ten additional eligible trials (−1.36%, −2.78 to +0.07; k = 15; *p* = 0.060). No analysis in this review demonstrated that the reflected wave was delayed. The phasic framework is therefore presented as an interpretation and hypothesis for prospective testing, and the phasic indices themselves remain critically under-reported.

## 1. Introduction

Heart failure with preserved ejection fraction (HFpEF) accounts for more than half of all heart failure hospitalisations worldwide and carries a prognosis comparable with that of heart failure with reduced ejection fraction [1], yet disease-modifying therapy remains sparse [2]. The prevailing explanatory model attributes HFpEF to a systemic pro-inflammatory state driven by comorbidities, which produces microvascular endothelial dysfunction, myocardial stiffening, and impaired relaxation [3]; a complementary account emphasises the haemodynamic consequences of impaired reserve [4]. Both reports account for the myocardium but say comparatively little about the vessel into which the myocardium ejects.

This vessel matters. Aortic stiffness predicts cardiovascular events and all-cause mortality independently of conventional risk factors [5], rising steeply with age and hypertension [6], and is now recognised as a determinant of the pulsatile load that the ventricle must overcome [7]. In HFpEF specifically, novel measures of arterial haemodynamics and wave reflection discriminate patients from controls better than blood pressure alone [8].

Classical ventriculo-arterial coupling models, built on end-systolic elastance (Ees) and effective arterial elastance (Ea), quantify load as a magnitude; that is, how much afterload the ventricle faces, averaged over the cardiac cycle [9]. This scalar framing is powerful but blind to timing. It cannot distinguish a stiff aorta that returns its reflected wave in late systole from an equally stiff aorta that returns it in diastole, even though the two impose entirely different burdens on the subendocardium [10].

The physiological basis for a timed account has long since been established. The two-element Windkessel model describes the aorta as a compliance that fills during ejection and discharges during diastole [11], and reservoir–wave separation formalises the decomposition of the pressure waveform into a reservoir component and a travelling wave component [12]. What has been missing is a clinical vocabulary that treats these as phases of the artery, synchronised with—or dissociated from—the phases of the ventricle [13]. We propose two such phases as follows.

*Arterial diastole*—The compliant reservoir-filling phase that occurs during LV systole, in which healthy elastic arteries expand to buffer the stroke volume, storing energy and limiting the systolic pressure excursion.

*Arterial systole*—The elastic recoil phase that occurs during LV diastole, in which the aorta releases stored energy, sustains diastolic pressure, and drives coronary perfusion.

**Figure 1:**
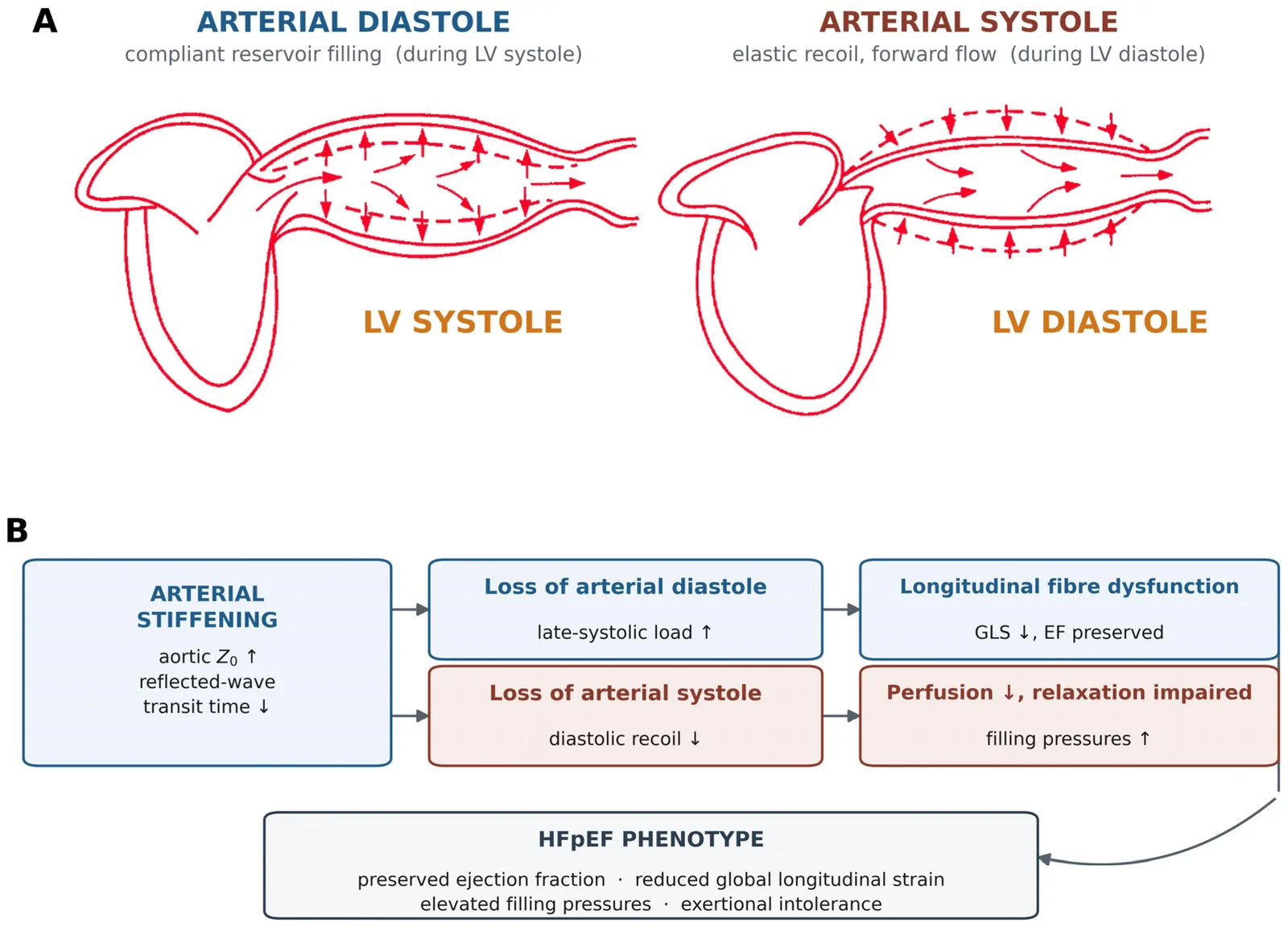
The phasic arterial framework. (**A**) Schematic of the two arterial phases. During LV systole, the compliant aorta distends to accommodate the stroke volume—arterial diastole; during LV diastole, it recoils, propelling the stored volume forward and sustaining diastolic and coronary perfusion pressure—arterial systole. (**B**) Mechanistic chain by which stiffening desynchronises the two phases and converges on the HFpEF phenotype. Z_0_ = characteristic aortic impedance; GLS = global longitudinal strain; EF = ejection fraction.

In this framework, the central pathological event of arterial stiffening is not an elevated pulse wave velocity per se but a temporal dyssynchrony between ventricular and arterial events. A premature return of the reflected wave truncates arterial diastole and loads the ventricle in late systole, where the helically arranged subendocardial longitudinal fibres are most vulnerable [14]; an attenuated recoil truncates arterial systole and withdraws perfusion pressure from those same fibres during the interval in which they are perfused. Both failures converge on the HFpEF phenotype: preserved global ejection fraction maintained by radial and circumferential shortening, with reduced global longitudinal strain (GLS) [15] and elevated filling pressures (Figure 1). Notably, neither of the two contemporary diagnostic algorithms for HFpEF incorporates any arterial variable [16,17].

The framework is testable. It first predicts that every domain of arterial stiffness should be elevated in HFpEF relative to controls without heart failure; second, that stiffness should track longitudinal myocardial dysfunction; third, that drug classes with documented arterial effects should measurably restore phase relationships; and fourth, that indices which explicitly encode timing—reflected-wave transit time, excess pressure, reservoir pressure—should discriminate more sharply than scalar stiffness measures. We therefore conducted a systematic review and meta-analysis to test these four predictions against the published evidence.

## 2. Materials and Methods

### 2.1. Protocol and Registration

This systematic review and meta-analysis was conducted and reported in accordance with the PRISMA 2020 statement [18] and the PRISMA-S extension for reporting literature searches [19]. The protocol was registered on the Open Science Framework (https://doi.org/10.17605/OSF.IO/FT6Q4); the full protocol, the screening register, the extraction database, and the analysis code are available within the project. The registration is retrospective with respect to data collection, which is declared in the registration itself and in Section 4.7. Deviations from the protocol are reported in Section 2.8. The dates of each stage of the review process are given in Table 1. The searches were run on a single day across all twelve queries; screening, full-text assessment, and data extraction followed sequentially, and the protocol was registered before the first pooled estimate was computed.

### 2.2. Eligibility Criteria

Eligibility followed a PECO structure defined separately for each of the three objectives.

**Objective 1 (observational).** Adults (≥18 years) with HFpEF, or with documented diastolic dysfunction and preserved ejection fraction, were compared with a control group without heart failure (healthy volunteers, hypertensive patients without heart failure, patients with non-cardiac dyspnoea, or subjects with normal diastolic function), in whom at least one arterial stiffness index was reported with a measure of central tendency and dispersion for both groups (Objective 1a); or in whom a correlation coefficient between an arterial stiffness index and GLS was reported (Objective 1b).

**Objective 2 (interventional).** Primary randomised controlled trials in adults with HFpEF, heart failure across the broader spectrum, or arterial hypertension, comparing a pre-specified drug class (angiotensin-converting enzyme inhibitors [ACEis] or angiotensin receptor blockers [ARBs]; angiotensin receptor-neprilysin inhibitors [ARNis]; sodium-glucose co-transporter 2 inhibitors [SGLT2is]; calcium channel blockers [CCBs]; organic nitrates; mineralocorticoid receptor antagonists [MRAs]) with placebo, standard care, or another drug class, all while reporting an arterial stiffness index as an outcome. No minimum treatment duration was imposed.

**Objective 3 (phasic indices).** Studies reporting temporal or phase-specific indices—reflected-wave transit time, excess pressure, reservoir pressure, pulse pressure amplification—in patients with heart failure, HFpEF, or diastolic dysfunction.

Accepted stiffness indices included carotid–femoral pulse wave velocities, measured and reported according to the expert consensus document [20] and interpretable against published reference values [21]; brachial–ankle and other regional pulse wave velocities; the augmentation index (AIx) and its heart-rate-corrected form (AIx@75); effective arterial elastance (Ea) and ventriculo-arterial coupling (Ea/Ees); aortic distensibility and strain; the cardio-ankle vascular index (CAVI); the beta stiffness index; central pressure and central haemodynamics; and pulmonary arterial stiffness or right ventricular–pulmonary arterial coupling. Exclusions were: ineligible publication types, including conference abstracts without full texts; reviews and meta-analyses; preclinical work; ineligible populations; no stiffness indices reported; the absence of randomisation or of a control group for Objective 2; ineligible interventions (non-pharmacological, a drug outside the pre-specified classes, or inorganic/dietary nitrate); no extractable data; retracted publications; and, for Objective 1, the absence of both an eligible comparator and a stiffness–GLS correlation.

### 2.3. Information Sources and Search Strategy

MEDLINE/PubMed, Scopus, and Web of Science were searched from inception to 10 September 2026 with no language restrictions. The search combined three conceptual axes: (A) HFpEF and diastolic dysfunction; (B) arterial mechanics (arterial stiffness, pulse wave velocity, wave reflection, augmentation index, reservoir–wave separation, ventriculo-arterial coupling); and (C) myocardial mechanics and the pre-specified pharmacological classes. Animal filters were applied within each module. Reference lists of included articles and of key monographs [13] were searched by hand. The complete line-by-line strategies for each database, reproduced exactly as they were run, are provided in Table S3 and in the OSF project.

### 2.4. Selection Process and Data Collection

Records were screened by their titles and abstracts against a written rubric, then by their full texts. Screening and extraction were performed in a single structured pass against that rubric and a pre-specified extraction schema, with large-language-model assistance and author adjudication, rather than by two reviewers working independently. Each decision and extracted value were recorded with its justification and the source it came from, and the complete screening register and extraction database are deposited in the OSF project, so that the procedure is open to audit. Each full text was read in its entirety, including its tables, against a pre-specified extraction schema. Extracted variables comprised the study design and setting; source cohort and trial registration number; population definition, including the ejection fraction threshold used, group sizes, and baseline characteristics; each reported stiffness and phasic index with its measurement method, unit, source table, and dispersion type; GLS, and its correlations with stiffness; adjusted associations; and, for randomised trials, the arms with the drug class and dose, as well as the baseline, final, and change values per arm and the between-arm differences with confidence intervals.

Two conventions governed extraction. First, no value was ever estimated; if a number was not printed explicitly in the text or a table, the field was left empty and the reason why recorded, such that values available only in figures are identified rather than approximated. Second, medians were not converted to means and standard errors were not converted to standard deviations at the point of extraction; the dispersion type was recorded verbatim, and conversions were later performed once under documented rules (Section 2.7). The five analysed datasets that result in one row per contrast, as used in the synthesis, are provided in Table S4.

### 2.5. Risk-of-Bias Assessment

Observational studies were assessed with the Newcastle–Ottawa Scale [22] using the cross-sectional adaptation where appropriate (selection, maximum 4; comparability, maximum 2; outcome, maximum 3). Randomised trials were assessed with the Cochrane RoB 2 tool [23] across the five domains—randomisation process, deviations from intended interventions, missing outcome data, measurement of the outcome, and selection of the reported result—with an overall judgement of low risk, some concerns, or high risk.

### 2.6. Handling of Overlapping Publications

Multiple reports of the same cohort or trial are a material threat to validity in the literature. Overlaps were detected automatically from the structured trial-registration fields (ClinicalTrials.gov, ISRCTN, UMIN, ACTRN, CTRI) and from the structured cohort-name field and were confirmed manually. In the originally submitted version, this matching also searched the free-text overlap annotation recorded for each study; because that annotation names other cohorts in order to deny membership—for example, “not a named epidemiological cohort (not MESA/ARIC/Framingham)”—the procedure treated unrelated studies as reports of the same cohort and excluded them. The procedure was corrected to read only the structured fields and the affected estimates were recomputed: the brachial–ankle pulse wave velocity moved from k = 5 to k = 7 and the cardio-ankle vascular index from k = 4 to k = 5, with both estimates strengthening. Where two or more reports shared participants, only one contributed to any given pooled estimate; the report with the largest analysed sample for that index was retained, while the remainder were kept in the evidence table but excluded from pooling. All overlap clusters, with the keys on which each was detected and the reports retained for each pooled estimate, are listed in Table S5.

### 2.7. Statistical Analysis

All syntheses used random-effects models with DerSimonian–Laird estimations of the between-study variance τ^2^ [24] and Hartung–Knapp–Sidik–Jonkman adjusted confidence intervals [25], which maintain nominal coverage better than the classical Wald interval when the number of studies is small. Pooling was performed only when at least three independent studies contributed to an index; indices were never pooled across families, and units were harmonised to a single scale before pooling. Where a trial reported more than one estimate for the same index—typically a whole-cohort result together with the results of age or severity subgroups—only the whole-cohort estimate at the trial’s primary time point entered the analysis, such that no participant contributed more than once to any pooled estimate. The primary effect measure for Objective 1a was the mean difference between the HFpEF and the control group, with Hedges’ g reported as a supporting standardised measure; for Objective 2, it was the mean difference in change from baseline between the pre-specified class arm and its comparator. Correlations (Objective 1b) were pooled after Fisher z transformation and back-transformed for presentation.

Heterogeneity was quantified with τ^2^, I^2^ [26], and Cochran’s Q, and a 95% prediction interval was computed whenever three or more studies contributed [27]. Leave-one-out analyses were run for four or more studies, whereas small-study effects were examined with funnel plots and Egger’s regression tests [28] when ten or more studies contributed. Pre-specified sensitivity analyses excluded, in turn, studies in which the extraction flagged ambiguity between standard deviation and standard error; studies requiring any numerical conversion; studies in populations affected by the protocol decision described in Section 2.8; and, for randomised trials, studies at an overall high risk of bias. Subgroup analyses by drug class were performed where at least three trials per class were available. The certainty of evidence was considered narratively against GRADE domains [29].

Conversions were applied under fixed rules and logged individually: medians with interquartile or full ranges were converted to means and standard deviations using the methods of Wan et al. [30] and Luo et al. [31]; standard errors were converted as SD = SE × √*n*; and half-widths of 95% confidence intervals were converted as SD = half-width × √n/1.96. Every conversion applied is listed individually with its arithmetic in Table S6. Analyses were performed in Python 3.11 [32]; the complete analysis code, the harvested datasets, and Stata do-files reproducing every figure are provided in the OSF project.

### 2.8. Protocol Decisions and Deviations

Four decisions were taken after the protocol was written and are reported here in full. First, the screening rubric admitted, for Objective 2, populations described as “hypertension or another cardiometabolic population”, whereas the analytic PECO admitted only HFpEF, heart failure across the broader spectrum, and hypertension. We resolved this in favour of the narrower analytic definition for the primary analysis, applying the rule that hypertension had to be an inclusion criterion rather than a comorbidity; the 46 studies in diabetes, chronic kidney disease, dialysis, peripheral arterial disease and coronary disease that this excluded were extracted in full and are reported in a sensitivity analysis. Second, where an eligible drug class appeared only as the comparator rather than as the experimental arm, the trial was retained, since the contrast still estimates the effect of that class relative to another agent. Third, head-to-head comparisons of two agents within the same pre-specified class were retained, and trials randomising a strategy rather than a drug class—identical agents in both arms differing only in target, schedule or dosing time—were excluded. Fourth, the experimental arm was defined as the first arm whose drug class belonged to one of the six pre-specified classes. Where both arms contained eligible classes, this rule assigns the direction of effect according to the order in which the arms are presented in the source article; we acknowledge that this choice is arbitrary, and all such comparisons are listed in Table S14. For within-class comparisons, no direction can be interpreted as favouring the pre-specified class, and we have avoided this wording throughout.

### 2.9. Data Verification

All values entering a pooled estimate—744 values from 83 studies, grouped into 138 contrasts—were reviewed individually by a second author (C.V.). For each contrast, the reviewer was shown the extracted value, the two groups under comparison, the unit, any transformations being applied, and the exact location of the value in the source article, and they recorded agreement or objection. Where the extracted value raised a question that agreement or disagreement could not close—the identity of a comparator, the label attached to a dispersion measure, or which of several reported estimates represented the whole randomised cohort—the question was posed in open form and answered in free text. This was a structured review with a recorded response for every value; it was not an independent duplicate extraction and is therefore not presented as one. The completed verification record is deposited in Table S18, and the findings it produced are reported in Section 3.3, Section 3.4, and Section 3.8 and summarised in Table S17.

## 3. Results

### 3.1. Study Selection

**Figure 2:**
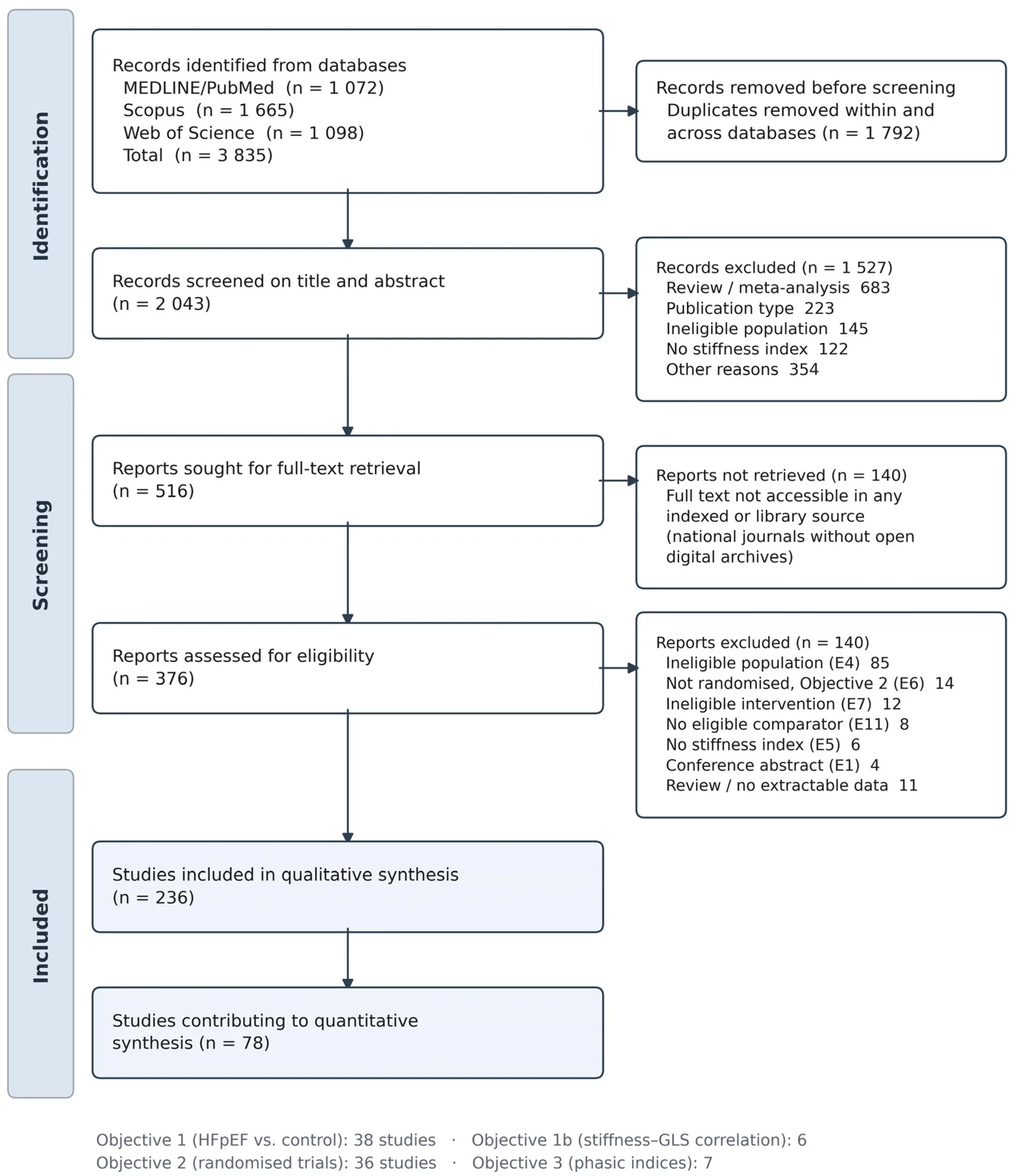
PRISMA 2020 flow diagram of study identification, screening, and inclusion. Exclusion codes for full-text screening: E1, conference abstract without full text; E4, ineligible population; E5, no arterial stiffness index; E6, no randomisation or no control group for Objective 2; E7, ineligible intervention; and E11, no eligible comparator and no stiffness–GLS correlation.

The search returned 3835 records (1072 from MEDLINE/PubMed, 1665 from Scopus and 1098 from Web of Science). After removal of 1792 duplicates within and across databases, 2043 records were screened by their titles and abstracts, of which 1527 were excluded. Full texts were sought for 516 reports; 140 could not be retrieved from any indexed or library source, almost all of which were articles in national journals without open digital archives. Of the 376 reports assessed in full, 140 were excluded, leaving 236 studies in the qualitative synthesis. Eighty-eight of these contributed at least one row to an analysis set, and 84 contributed to at least one pooled estimate; 81 of the 236 eligible studies, together with the three full texts, were recovered after peer review (Table S16). The per-study reason for non-contribution is given in Table S13 (Figure 2). Each excluded full text is listed with its code and the reason recorded at the time of the decision in Table S7.

### 3.2. Characteristics of Included Studies

Thirty-eight studies contributed to Objective 1a, comparing patients with HFpEF or diastolic dysfunction with preserved ejection fraction against controls without heart failure (Table 2). Designs were predominantly cross-sectional, with a smaller number of case–control studies and prospective cohorts. Six studies reported a correlation between an arterial stiffness index and a strain-derived measure of left ventricular function (Objective 1b), of which four were commensurate and contributed to the pooled estimate, and seven contributed phasic indices to Objective 3 (while 28 reported one, only these seven were against an eligible comparator in extractable form). Thirty-six randomised trials contributed to Objective 2 (Table 3); ARBs were the most frequently studied class, followed by CCBs, ACEis, and ARNis, with only isolated trials of SGLT2is and MRAs meeting the population criterion. All 236 studies included in the qualitative synthesis, with their designs, populations, and indices they report, are listed in Table S8.

### 3.3. Objective 1a: Arterial Stiffness in HFpEF Versus Controls

**Figure 3:**
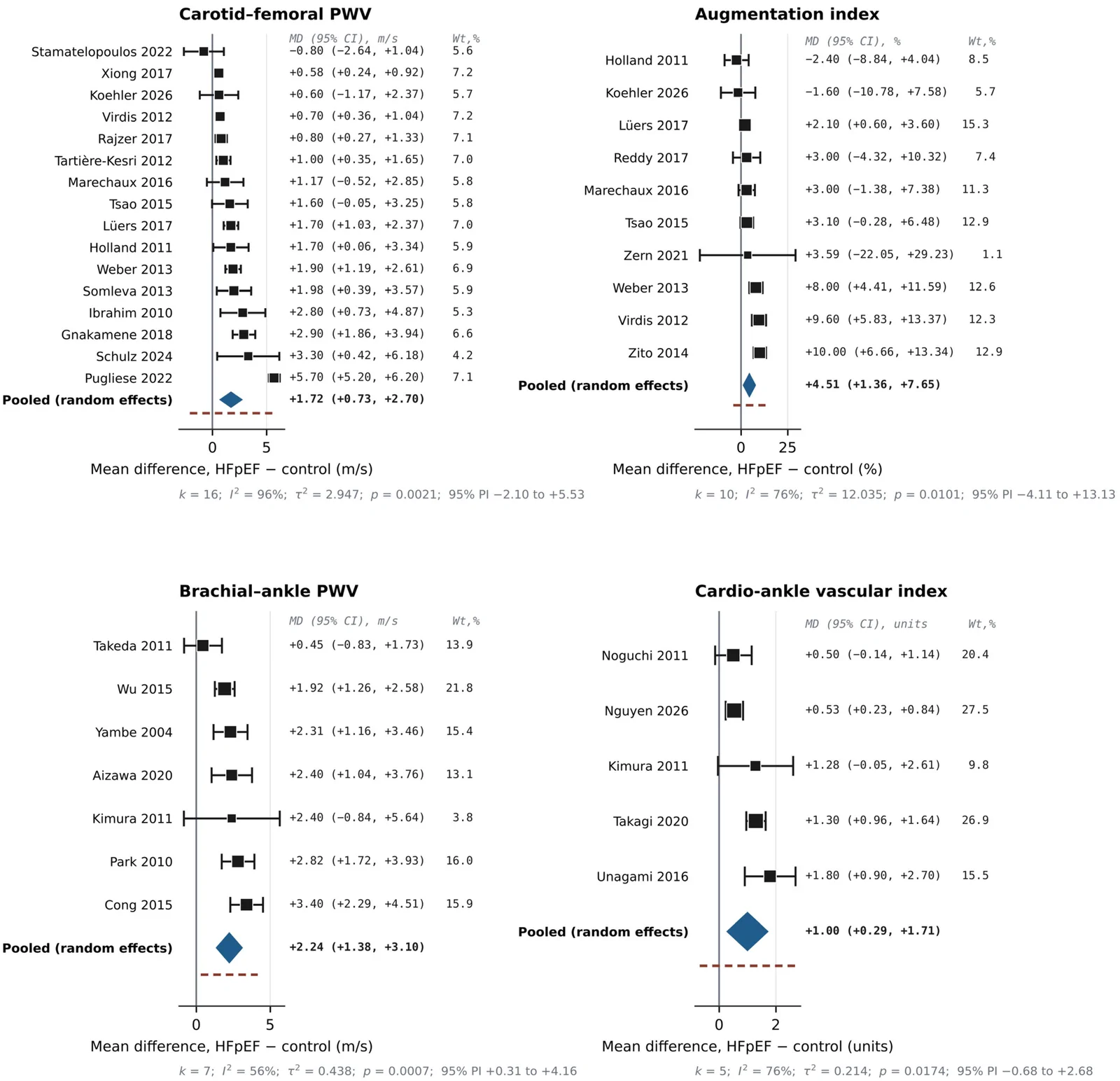
Forest plots for Objective 1a—arterial stiffness in HFpEF or diastolic dysfunction with preserved ejection fraction versus control without heart failure. Squares are study mean differences scaled by random-effects weight, horizontal lines are 95% confidence intervals, the diamond is the pooled estimate, and the dashed line beneath it is the 95% prediction interval. Positive values indicate greater stiffness in HFpEF. Carotid–femoral pulse wave velocity includes 16 studies and brachial–ankle pulse wave velocity includes seven following full-text recovery and the correction to the overlap procedure described in Section 2.6.

Every stiffness domain that reached the pooling threshold was higher in HFpEF than in controls (Figure 3, Table 4). Carotid–femoral pulse wave velocity was +1.72 m/s (95% CI +0.73 to +2.70) higher across 16 studies and 3095 participants (*p* = 0.002), with I^2^ = 96%, τ^2^ = 2.947. Brachial–ankle pulse wave velocity was +2.24 m/s (95% CI +1.38 to +3.10) higher across seven studies (*p* = 0.0007, I^2^ = 56%), while the augmentation index was +4.51% (95% CI +1.36 to +7.65) higher (10 studies, *p* = 0.010) and the cardio-ankle vascular index +1.00 (95% CI +0.29 to +1.71) higher (five studies, *p* = 0.017). The augmentation index, corrected to 75 beats per minute, reached the pooling threshold for the first time after full-text recovery and did not differ (+2.25%, 95% CI −1.10 to +5.59; k = 3; *p* = 0.102; I^2^ = 0%). Effective arterial elastance was numerically higher, but neither the mean difference (+0.187 mmHg/mL (95% CI −0.161 to +0.535), *p* = 0.237) nor the standardised difference (Hedges’ g +0.33 (95% CI −0.12 to +0.78), *p* = 0.122) reached significance. Effective arterial elastance indexed to the body surface area, a distinct quantity that was pooled separately, also did not differ (+0.12 mmHg·m^2^/mL (95% CI −0.31 to +0.56), k = 3, *p* = 0.340). Ventriculo-arterial coupling, expressed as Ea/Ees, did not differ (+0.056 (95% CI −0.320 to +0.433), *p* = 0.699) despite extreme heterogeneity (I^2^ = 98%, τ^2^ = 0.029). This heterogeneity is not random noise. The extremes of the cfPWV analyses are a cardiopulmonary-exercise cohort in which patients exceeded controls by 5.7 m/s [53] and a cohort in which the point estimate was negative [57]; between them lie the magnetic-resonance-derived estimates [56] and tonometric estimates that are not numerically interchangeable. The prediction intervals are correspondingly wide and, for five of the six pooled indices, include no differences; brachial–ankle pulse wave velocity is the exception (+0.31 to +4.16 m/s), and it is also the index with the lowest residual heterogeneity. This is the single most important qualification of these results; although the average HFpEF cohort has stiffer arteries than the average control cohort, the magnitude of that difference is not transportable to a new setting with any precision [27].

### 3.4. Objective 1b: Arterial Stiffness and Global Longitudinal Strain

Six studies [36,50,56,107,108,109], totalling 469 participants, reported a correlation coefficient between an arterial stiffness index and a strain-derived measure of left ventricular function. Independent verification showed that in two of them the second variable was not a measure of longitudinal function—in one, the ratio of endocardial to epicardial systolic strain, while in the other, the circumferential peak early diastolic strain rate—and these were excluded as non-commensurate. Pooling the four remaining studies gave r = −0.28 (95% CI −0.65 to +0.20; *p* = 0.162), with I^2^ = 79%. The direction of the reported coefficients was inconsistent across studies, and the confidence intervals span both moderate negative and moderate positive associations. The prediction of the phasic framework that stiffness should track longitudinal dysfunction is therefore not supported by the pooled evidence; however, with only four commensurate studies, this is an absence of evidence rather than evidence of absence, and it identifies the single largest gap in the field.

### 3.5. Objective 2: Pharmacological Modification of Arterial Phase

Thirty-six randomised trials contributed. The pre-specified drug classes reduced heart-rate-corrected augmentation index relative to their comparators by −2.18% (95% CI −3.52 to −0.85) across 11 trials and 1432 participants (*p* = 0.004; I^2^ = 73%, τ^2^ = 1.359). In contrast, the carotid–femoral pulse wave velocity was essentially unchanged (−0.11 m/s (95% CI −0.40 to +0.18); 18 trials; *p* = 0.428), as were the brachial–ankle pulse wave velocity (−0.56 m/s (95% CI −1.40 to +0.28); *p* = 0.165) and uncorrected augmentation index (−1.32% (95% CI −6.48 to +3.83); *p* = 0.586), the latter of which exhibiting essentially complete heterogeneity (I^2^ = 100%, τ^2^ = 69.277).

**Figure 4:**
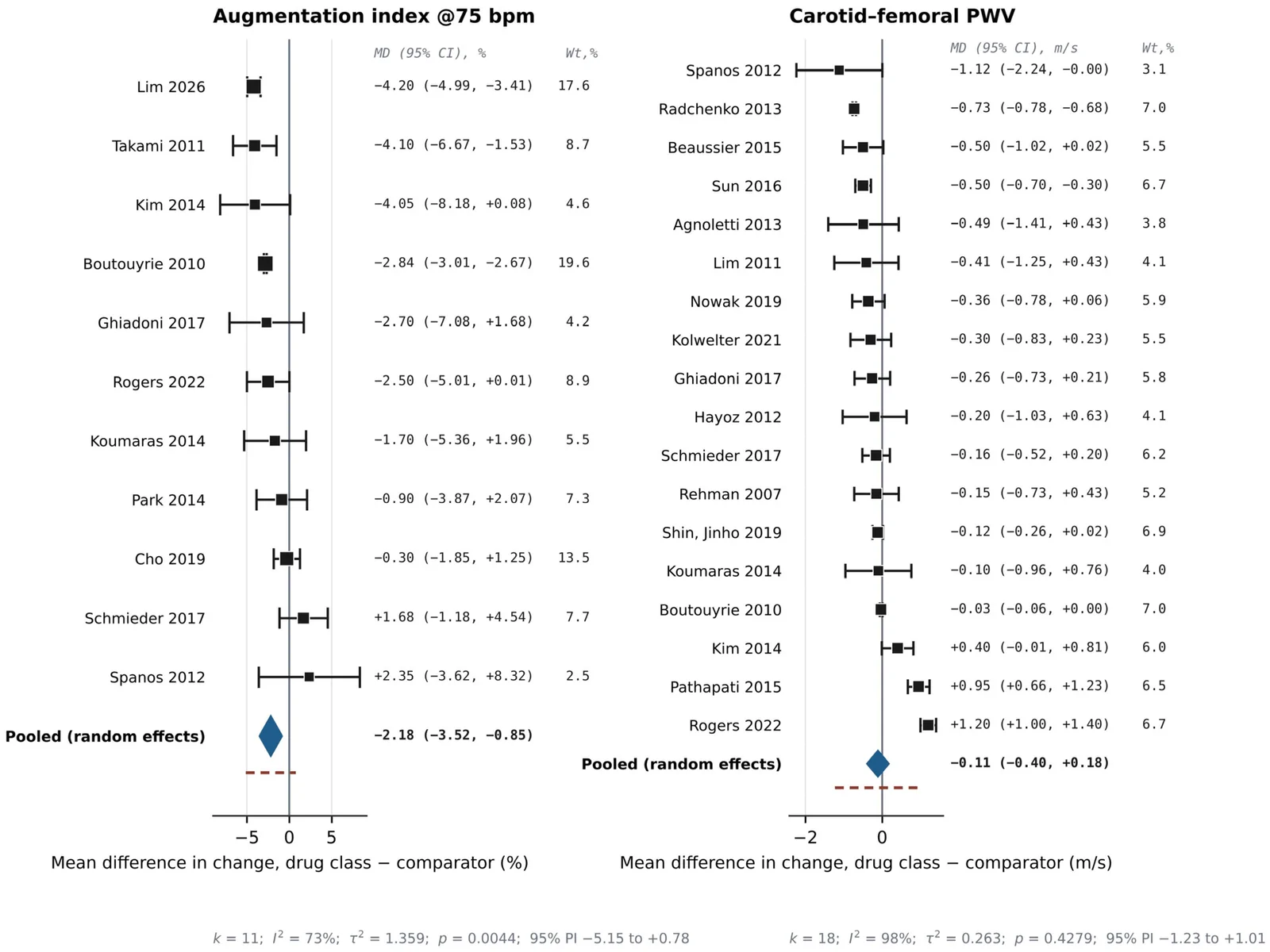
Forest plots for Objective 2—effect of pre-specified drug classes on arterial stiffness in randomised trials. Negative values favour the pre-specified class over its comparator. The dissociation between a significant reduction in heart-rate-corrected augmentation index and a null effect on carotid–femoral pulse wave velocity is the central result of this synthesis. Squares are study mean differences scaled by random-effects weight, horizontal lines are 95% confidence intervals, the diamond is the pooled estimate, and the dashed line beneath it is the 95% prediction interval.

The AIx@75 result is driven by trials that are consistent in direction. The largest individual reductions came from a comparison between nebivolol with telmisartan [84], an azelnidipine trial [94], and a losartan–carvedilol comparison [83], each around four percentage points; the largest trial, a 393-patient comparison of amlodipine–valsartan with amlodipine–atenolol [75], contributed a reduction of 2.84% and carries the greatest weight. Only two trials point the other way, a sacubitril/valsartan versus olmesartan comparison [97] and an aliskiren versus valsartan comparison [92], with both having wide intervals. The heterogeneity of the uncorrected AIx analysis, by contrast, is largely artefactual: two trials report dispersions that are almost certainly standard errors mislabelled as standard deviations, and excluding them reduces I^2^ from 100% to 88% (Section 3.8, Figure 4).

Only one class was represented by enough trials for a subgroup estimate. ARB reduced AIx@75 by −2.58% (95% CI −4.04 to −1.12; k = 8; *p* = 0.004). For every other class the number of eligible trials fell below the pre-specified threshold of three, so no class-specific estimate could be computed and the planned meta-regression across classes was not performed. This is a finding in its own right: the pharmacological literature on arterial stiffness is dominated by renin–angiotensin blockade, and the classes with the strongest recent outcome evidence in HFpEF [110,111,112] have almost no randomised arterial-mechanics data in an eligible population.

Two further analyses bear on the robustness of this dissociation. Excluding trials at an overall high risk of bias, the reduction in AIx@75 was confirmed (−2.28%, 95% CI −3.79 to −0.76; k = 9; *p* = 0.008), whereas carotid–femoral pulse wave velocity, which was unchanged in the primary analysis, became significant (−0.21 m/s, 95% CI −0.41 to −0.02; k = 13; *p* = 0.036), with I^2^ falling from 98% to 64%. Separately, ten eligible randomised trials reported only the between-arm difference with its confidence interval and could not contribute under the pre-specified effect measure; adding them by generic inverse variance reduced the pooled AIx@75 effect to −1.36% (95% CI −2.78 to +0.07; k = 15; *p* = 0.060). The only significant pharmacological result of this synthesis therefore does not survive a reasonable extension of the inclusion rule.

### 3.6. Objective 3: Phasic Indices

Twenty-eight eligible studies reported at least one phasic index, but only seven reported it against an eligible comparator in a form that could be extracted [43,47,48,55,57,63,113]. Reflected-wave transit time, the index that most directly operationalises the framework, was reported in milliseconds by four of these and was −5.09 ms (95% CI −11.45 to +1.27) shorter in patients than in controls (*p* = 0.086; I^2^ = 0%, τ^2^ = 0.000). Two further studies reported the same construct on non-commensurate scales—as a percentage of ejection time and in ms^2^—and were therefore not pooled with them. The point estimate is in the direction the framework predicts—an earlier wave return—but the confidence interval crosses zero. Excess pressure, reservoir pressure, and pulse pressure amplification were each reported by a single eligible study and could not be pooled.

The scarcity is itself the result. Of 236 eligible studies, 28 (11.9%) reported any phasic index at all and only 17 (7.2%) reported one that encodes the timing of the reflected wave, even though reservoir–wave separation has been available for two decades [12] and requires no equipment beyond a tonometer. Fewer still reported it in a form that permitted pooling against a comparator.

### 3.7. Risk of Bias and Small-Study Effects

Among the 36 randomised trials contributing to Objective 2 (Table 3), one was judged at a low overall risk of bias, 23 raised some concerns and 12 were at high risk [23], most commonly because of open-label design with unblinded outcome assessment, per-protocol rather than intention-to-treat analysis, and post hoc rather than pre-specified reporting of the arterial endpoint. Thirty-six of the 38 observational studies of Objective 1a could be scored on the Newcastle–Ottawa Scale [22] and achieved a median of six (range 2–8), with comparability the most frequent weakness; across the whole eligible corpus, 105 observational studies were scored and 223 randomised trials were assessed with RoB 2, and both sets of judgements are reported in full, by domain, in Tables S9 and S10.

**Figure 5:**
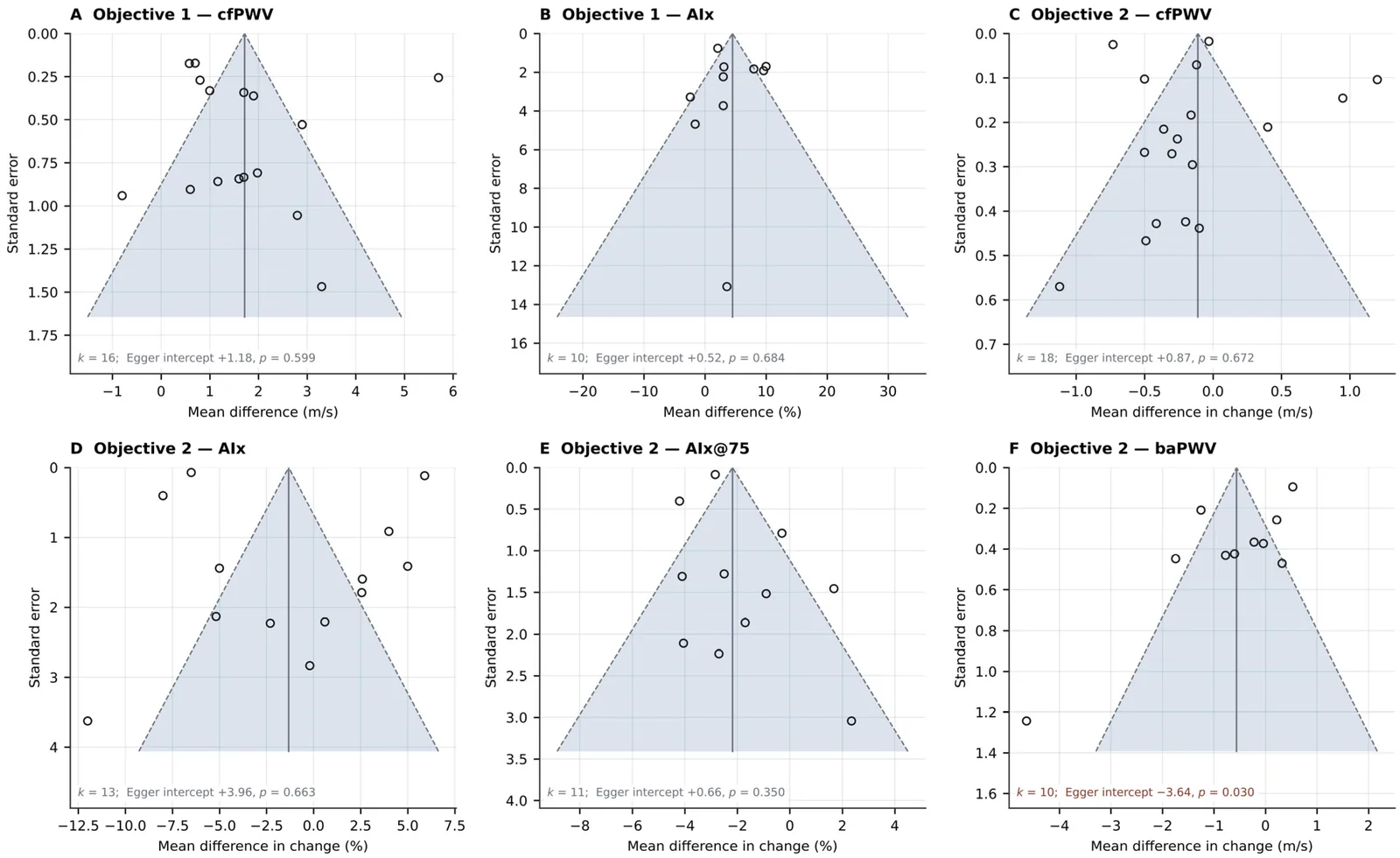
Funnel plots for all pooled indices with at least ten contributing studies. The vertical line is the pooled estimate and the shaded triangle the pseudo-95% confidence region; Egger’s regression intercept and its *p* value are given in each panel. (**A**) Objective 1a, carotid–femoral pulse wave velocity. (**B**) Objective 1a, augmentation index. (**C**) Objective 2, carotid–femoral pulse wave velocity. (**D**) Objective 2, augmentation index. (**E**) Objective 2, heart-rate-corrected augmentation index. (**F**) Objective 2, brachial–ankle pulse wave velocity—the only index showing significant funnel asymmetry. The dashed lines are the pseudo-95% confidence limits; red type marks the only panel with significant funnel asymmetry.

Funnel plots and Egger’s tests [28] were computed for the indices with at least ten contributing studies (Figure 5). There was no statistical evidence of small-study effects for cfPWV in Objective 1a (intercept +1.18, *p* = 0.599), for the augmentation index in Objective 1a (+0.52, *p* = 0.684), for cfPWV in Objective 2 (+0.87, *p* = 0.672), for the augmentation index in Objective 2 (+3.96, *p* = 0.663), or for AIx@75 in Objective 2 (+0.66, *p* = 0.350). Egger’s test was significant for brachial–ankle pulse wave velocity in Objective 2 (intercept −3.64, *p* = 0.030), which should be interpreted cautiously given that asymmetry with this degree of heterogeneity may reflect true between-study differences rather than publication bias [26].

### 3.8. Sensitivity Analyses

The principal results were robust to the pre-specified sensitivity analyses (Table 5), with two informative exceptions. Excluding the nine Objective 1a studies, in which the extraction flagged ambiguity between standard deviation and standard error, left the cfPWV estimate essentially unchanged but rendered the augmentation index estimate non-significant, indicating that the AIx result rests partly on studies whose dispersion reporting could not be verified. Conversely, the Objective 2 AIx@75 result strengthened under every restriction: excluding studies that required conversions gave a larger effect with a narrower interval, and excluding trials at a high risk of bias left it intact (−2.28%, k = 9, *p* = 0.008). The same restriction, however, changed the carotid–femoral result in Objective 2 from null to significant (−0.21 m/s, k = 13, *p* = 0.036), with heterogeneity falling from 98% to 64%, indicating that much of the dispersion in the primary estimate arose from trials at high risk of bias. By restricting this to studies unaffected by the cardiometabolic population decision changed no estimate because none of the affected studies contributed to a pooled analysis under the narrower definition. Leave-one-out analyses for each pooled estimate with four or more contributing studies are given in Table S11.

## 4. Discussion

This synthesis establishes three things. Table 6 states, for each of the four pre-specified predictions, whether the analysis supported it, did not support it, or could not test it, together with the numerical basis for that judgement. First, the association between HFpEF and arterial stiffness is real and consistent in terms of direction across every measurement domain that could be pooled, which extends to a formal meta-analysis what individual cohorts have reported for two decades [5,8]. Second, that association is heterogeneous to a degree that forbids treating any pooled mean as a clinical constant. Third—and this is the finding that bears on the framework—the component of arterial load that responds to current pharmacotherapy is not the component that is most elevated in HFpEF.

### 4.1. Dissociation Between Conduit Stiffness and Wave Reflection

Randomised trials of the pre-specified drug classes reduced heart-rate-corrected augmentation index by roughly two percentage points relative to comparators, yet moved carotid–femoral pulse wave velocity not at all. Since AIx@75 is a composite of reflected wave amplitude and timing while cfPWV is a measure of conduit wall stiffness [20], this dissociation says that these drugs act on the reflection properties of the arterial tree rather than on the structural stiffness of the aorta. In the phasic vocabulary, this is a change in the amplitude of wave reflection. Whether it is accompanied by a change in the timing of the returning wave is not established by these data; AIx@75 is a composite quantity, influenced by the amplitude and the timing of reflection but also by heart rate, stroke volume and the shape of the incident wave, and the only analysis addressing timing directly—reflected-wave transit time—rests on four studies and does not differ between groups (−5.09 ms, 95% CI −11.45 to +1.27; *p* = 0.086).

The observation is not without precedent. CAFE showed, within ASCOT, that two regimens equivalent on brachial pressure differed substantially in central aortic pressure and in clinical outcome [114], and the mechanistic literature has long held that wave reflection and conduit stiffness are separable determinants of the central waveform [7,10]. What the present synthesis adds is the quantitative asymmetry, where pooled across 18 randomised comparisons, cfPWV does not move at all over the durations studied, while AIx@75 does so reproducibly across 11 comparisons.

This has a direct methodological implication. If the arterial property that these drugs actually modify is wave reflection rather than aortic stiffness, then mechanistic trials in HFpEF should be powered on reflection-based endpoints, and on the timing indices that would distinguish a change in amplitude from a change in phase, rather than on pulse wave velocity. Identifying a therapeutic target in reflected-wave timing would require such trials; it is not a conclusion that the present synthesis can support. It also offers a partial explanation for why the large stiffness differences seen in cross-sectional HFpEF cohorts have not translated into treatment effects: trials such as NEAT-HFpEF, which tested an organic nitrate and found reduced activity rather than benefit [115], and PARAGON-HF, which narrowly missed its primary endpoint [116], were not designed around the arterial property that their drugs actually modify.

### 4.2. The Missing Evidence on Timing

The most striking result of this review is negative. The framework predicts that indices encoding phase—reflected-wave transit time, excess pressure, and reservoir pressure—should discriminate patients from controls more sharply than scalar stiffness. We could not test that prediction properly; although 28 of 236 eligible studies reported some phasic index, only seven did so against an eligible comparator in an extractable form [43,47,48,55,57,63,113], and only one index in one unit reached the pooling threshold. The pooled reflected-wave transit time was shorter in patients as predicted, but the interval crossed zero.

This is not a limitation of the framework; it is a description of the literature. Reservoir–wave separation has been available since the early 2000s [12] and imposes no additional measurement burden on a study already performing tonometry. Where it has been applied in a randomised setting the signal is informative; in the J-CORE trial, adding a calcium channel blocker rather than a diuretic to an ARB-lengthened reflected-wave transit time and reduced pulse pressure amplification, with between-arm differences that were statistically significant [117]. That a single trial can demonstrate a phase effect while the remainder of the literature is silent on it is the most readily remediable gap identified here.

### 4.3. Longitudinal Strain and the Stiffness–GLS Link

The pooled correlation between arterial stiffness and longitudinal strain was null, with wide confidence limits and severe heterogeneity across only four commensurate studies [36,50,107,109]; two further studies reported a correlation with a different construct and were excluded. The mechanistic argument linking late systolic load to selective longitudinal dysfunction—subendocardial longitudinal fibres bear the greatest wall stress and receive the least perfusion reserve [14]—remains physiologically sound, and the prognostic importance of reduced GLS in HFpEF despite preserved ejection fraction is firmly established [15]. What is missing is a body of studies that report the correlation in a comparable form. Studies routinely measure both GLS and a stiffness index and then report neither their joint distribution nor their correlation, so that the link that the framework predicts cannot be assessed even though the data to assess it have already been collected.

### 4.4. Pharmacological Classes and Phasic Targets

Table 7 maps the pre-specified classes onto the two arterial phases as the present data permit. The asymmetry of the evidence base is stark: renin–angiotensin blockade accounts for the majority of eligible randomised arterial data, while the two classes with the strongest recent outcome evidence in HFpEF—SGLT2 inhibitors [110,111] and mineralocorticoid receptor antagonists [112]—contributed only isolated trials to the eligible population, because their arterial-mechanics trials were overwhelmingly conducted in type 2 diabetes or chronic kidney disease rather than in hypertension or heart failure. Closing that gap requires no new drug, as it requires measuring tonometry in the populations in which these agents are already being tested.

### 4.5. Multimodality Assessment of Phasic Arterial Function

**Figure 6:**
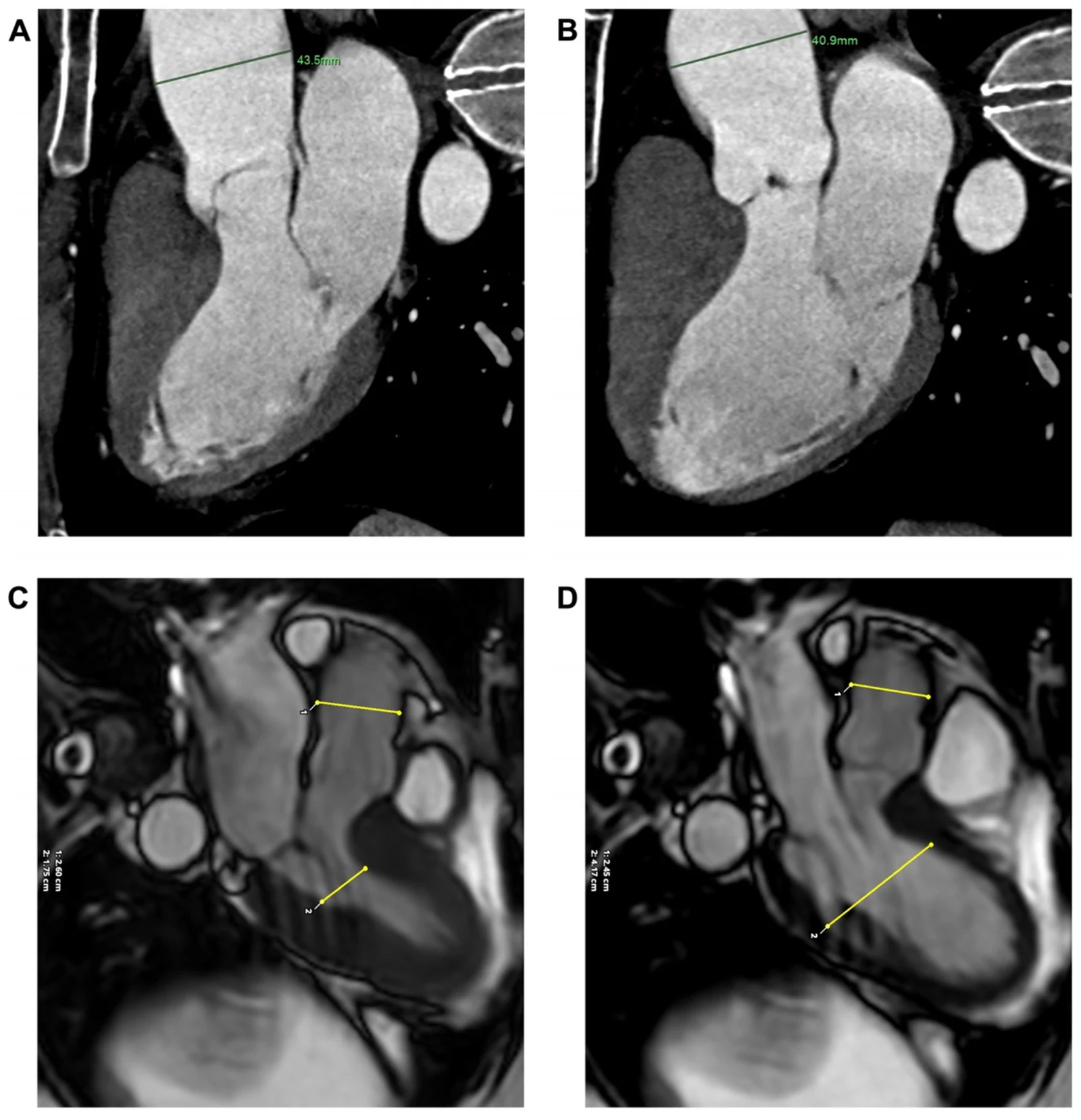
Phasic aortic dimension change assessed by multimodality imaging. (**A**,**B**) Electrocardiogram-gated computed tomography angiography of the same patient showing the ascending aortic diameter in systole ((**A**), 43.5 mm) and in diastole ((**B**), 40.9 mm); the systolic-to-diastolic difference is the geometric correlate of reservoir filling. (**C**,**D**) Cine cardiovascular magnetic resonance in a three-chamber view, with aortic and left ventricular dimensions measured at end-diastole (**C**) and end-systole (**D**). The proportional change in aortic cross-section over the cycle is the imaging expression of arterial diastole and arterial systole.

Phasic arterial function is directly visible with routine imaging. Electrocardiogram-gated computed tomography angiography and cine cardiovascular magnetic resonance resolve the aortic root and ascending aorta across the cardiac cycle, so that the systolic-to-diastolic change in cross-sectional area—the geometric expression of reservoir filling and recoil—can be measured in the same study that assesses chamber volumes (Figure 6). Ascending aortic strain derived this way is an emerging marker of ventriculo-arterial coupling [118] that requires no additional acquisition beyond what is already performed, and it offers a route to quantifying arterial systole and diastole in patients in whom tonometry is impractical. One of the studies pooled here derived aortic stiffness from cardiac magnetic resonance rather than tonometry [56], which the approach generalises.

### 4.6. Clinical and Research Implications

Three implications follow. First, arterial variables belong in the diagnostic assessment of HFpEF. Neither HFA-PEFF nor H_2_FPEF includes one [16,17], yet tonometry adds minutes to an echocardiographic study and the differences documented here are of the order of 1.7 m/s in cfPWV—larger than the increment associated with a decade of ageing in reference populations [21]. Second, mechanistic trials should be designed around reflection and timing. Third, sex-specific analyses deserve attention: women with HFpEF display a distinctly arterial phenotype, with higher augmentation index relative to body size and earlier wave reflection [119], and the present data were too sparsely stratified by sex to test whether the phase dissociation differs between men and women.

### 4.7. Limitations

Several limitations bound these conclusions. Heterogeneity ranged from substantial to extreme for most comparisons, and the prediction intervals cross the null for five of the six pooled indices [27]; the pooled means describe the average of a heterogeneous literature rather than an expected value for a new cohort. The definition of HFpEF varied across studies, and our protocol decision to count documented diastolic dysfunction with preserved ejection fraction as eligible widens the population further. 140 full texts could not be retrieved, concentrated in national journals without digital archives; thirteen of these were in the observational core, where each loss reduces k directly; seven of the thirteen were recovered after peer review and three entered the quantitative synthesis (Table S16). A substantial number of eligible studies reported their key values only in figures and were therefore unable to contribute numerically. Ambiguity between standard deviation and standard error was pervasive and, as the sensitivity analysis shows, materially affects the augmentation index result for Objective 1a. Measurement methods were not interchangeable: augmentation index measured as the P2/P1 ratio occupies a different numerical scale from the classical AP/PP index, and we kept the two apart rather than pooling them. Finally, the narrower population definition adopted for the primary analysis excludes a large and growing body of randomised arterial-mechanics data generated in diabetes and chronic kidney disease; those studies are extracted and reported, and a future synthesis adopting the broader definition would be substantially better powered for the newer drug classes. Certainty of evidence was assessed across the five GRADE domains for every principal pooled outcome (Table 8) [29]: low for carotid–femoral pulse wave velocity, augmentation index and brachial–ankle pulse wave velocity in Objective 1a, low for AIx@75 in Objective 2 with additional downgrading for indirectness because all contributing trials were conducted in hypertension, and very low for Objectives 1b and 3. Screening and extraction were performed in a single structured pass against a written rubric and a fixed schema, with large-language-model assistance and author adjudication, rather than by two independent reviewers. This procedure is more auditable than conventional dual screening, whose reconciliation typically leaves no record—every decision, every value and every source is deposited—but it is less robust against a systematic misreading that the rubric itself failed to anticipate. Readers should weigh it accordingly, and the deposited materials are provided so that they can.

Certainty was rated from the GRADE starting point for observational evidence (low) for Objectives 1a, 1b, and 3, and from the starting point for randomised evidence (high) for Objective 2, which was then downgraded as shown.

Three further limitations emerged from the revision. The analysis restricted to studies using a clinical or haemodynamic definition of HFpEF is fragile; the carotid–femoral estimate sits at *p* = 0.050 with a lower confidence limit of 0.004 m/s and does not withstand leave-one-out analysis, so the question is better regarded as unresolved for rigorously defined HFpEF than as answered. In one contributing study, the comparator is itself a disease stage—symptomatic (stage C) versus preclinical, asymptomatic (stage B) HFpEF—and verification confirmed that the article provides no disease-free comparison group; the affected estimates are reported both with and without that study. Finally, the verification described in Section 2.9 was a structured review in which the second author saw the extracted values; it can detect implausible values, mislabelled dispersion measures, and mismatched comparators, which it did so, but it cannot estimate an extraction error rate in the way that a blinded duplicate extraction would.

## 5. Conclusions

Reframing arterial stiffness as a disorder of timing rather than of magnitude is not a relabelling exercise: it makes predictions that the present synthesis can partly adjudicate. Patients with HFpEF have stiffer arteries per every pooled measure, albeit with heterogeneity that forbids treating any pooled mean as a clinical constant, and the arterial property that current pharmacotherapy is moving toward is wave reflection rather than aortic conduit stiffness. Three qualifications bound that statement: the reduction in reflection amplitude derives entirely from hypertensive populations and does not survive the inclusion of ten further eligible trials; carotid–femoral pulse wave velocity, unchanged in the primary pharmacological analysis, falls significantly when trials at a high risk of bias are excluded; and in rigorously defined HFpEF, the available data are insufficient to establish whether a stiffness difference exists. The phasic framework is therefore an interpretation and a hypothesis for prospective testing, and it argues for designing mechanistic trials in HFpEF around reflection-based and timing-based endpoints rather than pulse wave velocity.

The framework’s central prediction—that indices encoding phase discriminate better than scalar stiffness—remains untested because the literature almost never reports them. Reflected-wave transit time, excess pressure and reservoir pressure can be derived from measurements that thousands of published studies have already made. Reporting them would cost nothing and would allow the hypothesis advanced here to be confirmed or refuted within a single cycle of secondary analyses.

## Figures and Tables

**Table 1:** Dates of the review process.

Stage	Date
Searches across all twelve queries	10 September 2026
Title and abstract screening	10 September 2026
Full-text assessment	12 September 2026
Data extraction	September 2026
First pooled estimate	September 2026
Protocol registration on the Open Science Framework	September 2026
Co-author verification of extracted values	20 September 2026

**Table 2:** Characteristics of the 38 studies contributing to Objective 1a (HFpEF or diastolic dysfunction with preserved ejection fraction versus control without heart failure). N = combined sample of the two contrasted groups; NOS = Newcastle–Ottawa Scale total score.

Study	Country	Design	N	Stiffness Indices	NOS	Ref.
Borlaug (2010)	USA	Cross-sectional	31	Ea, Ea/Ees	6	[33]
Cong (2015)	China	Cross-sectional	111	baPWV	5	[34]
Fujimoto (2012)	USA	Prospective cohort	20	Ea, Ea/Ees	3	[35]
Gnakamene (2018)	France	Cross-sectional	65	cfPWV	6	[36]
Holland (2011)	Australia	Case–control	30	AIx, cfPWV	6	[37]
Ibrahim (2010)	USA	Cross-sectional	10	cfPWV	2	[38]
Ikonomidis (2008)	Greece	Cross-sectional	163	AIx (P2/P1), Aortic strain	7	[39]
Karagodin (2017)	USA	Case–control	77	Aortic distensibility, Ea, Aortic strain	5	[40]
Kim (2014)	South Korea	Cross-sectional	287	AIx (P2/P1)	7	[41]
Kimura (2011)	Japan	Cross-sectional	53	CAVI, baPWV	5	[42]
Koehler (2026)	Germany	Prospective cohort	51	AIx, cfPWV	6	[43]
Kosmala (2016)	Poland	Cross-sectional	267	Ea, Ea/Ees	7	[44]
Kresoja (2021)	Germany	Retrospective cohort	164	Aortic distensibility	6	[45]
Lekavich (2017)	USA	Case–control	118	Ea, Ea/Ees	6	[46]
Lüers (2017)	Germany	Cross-sectional	257	AIx, AIx@75, cfPWV	6	[47]
Marechaux (2016)	France	Case–control	90	AIx, cfPWV	6	[48]
Nguyen (2026)	Japan	Cross-sectional	335	CAVI	6	[49]
Noguchi (2011)	Japan	Cross-sectional	75	CAVI	4	[50]
Park (2010)	South Korea	Cross-sectional	62	baPWV	6	[51]
Popovic (2023)	USA	Retrospective cohort	468	Ea	8	[52]
Pugliese (2022)	Italy	Cross-sectional	259	Ea/Ees, cfPWV	8	[53]
Rajzer (2017)	Poland	RCT (substudy)	126	cfPWV	—	[54]
Reddy (2017)	USA	Cross-sectional	120	AIx, Ea	6	[55]
Schulz (2024)	Germany	Post hoc analysis	34	cfPWV	7	[56]
Stamatelopoulos (2022)	Greece	Case–control	48	AIx@75, cfPWV	5	[57]
Takagi (2020)	Japan	Case–control	200	CAVI	7	[58]
Takeda (2011)	Japan	Cross-sectional	337	baPWV	5	[59]
Tartière-Kesri (2012)	France	Case–control	38	Ea, Ea/Ees, cfPWV	6	[60]
Tsao (2015)	USA	Prospective cohort	272	AIx, cfPWV	8	[61]
Unagami (2016)	Japan	Cross-sectional	61	CAVI	5	[62]
Virdis (2012)	Italy	RCT (substudy)	50	AIx, cfPWV	—	[63]
Weber (2013)	Austria	Cross-sectional	136	AIx, cfPWV	6	[64]
Wu (2015)	China	Prospective cohort	758	baPWV	8	[65]
Xiong (2017)	China	Cross-sectional	1599	cfPWV	7	[66]
Yambe (2004)	Japan	Cross-sectional	147	baPWV	6	[67]
Yip (2011)	China (Hong Kong)	Case–control	172	Ea, Ea/Ees	8	[68]
Zern (2021)	USA	Cross-sectional	282	AIx, Ea	7	[69]
Zito (2014)	Italy	Cross-sectional	127	AIx	6	[70]

AIx = augmentation index; AIx@75 = augmentation index corrected to 75 beats per minute; baPWV = brachial–ankle pulse wave velocity; CAVI = cardio-ankle vascular index; cfPWV = carotid–femoral pulse wave velocity; Ea = effective arterial elastance; Ees = end-systolic elastance. A dash indicates that the item was not reported.

**Table 3:** Randomised controlled trials contributing to Objective 2, ordered by pre-specified drug class. N = combined sample of the two contrasted arms.

Trial	Class	Registration	N	Indices	RoB 2	Ref.
Ghiadoni (2017)	ACEi	EudraCT 2009-016738-28	76	AIx@75, cfPWV	Some concerns	[71]
Koumaras (2014)	ACEi	—	38	AIx, AIx@75, cfPWV	Some concerns	[72]
Tsang (2006)	ACEi	—	21	AIx	High	[73]
Bokuda (2010)	ARB	—	53	CAVI	High	[74]
Boutouyrie (2010)	ARB	NCT00687973	393	AIx, AIx@75, cfPWV	Some concerns	[75]
Cho (2019)	ARB	NCT02294539	187	AIx@75	Some concerns	[76]
Farag (2018)	ARB	—	137	AIx	High	[77]
Hayoz (2012)	ARB	—	109	cfPWV	Some concerns	[78]
Hong (2011)	ARB	NCT00599885	71	baPWV	Some concerns	[79]
Huang (2024)	ARB	NCT02893358	320	baPWV	Some concerns	[80]
Ishii (2008)	ARB	—	22	baPWV	High	[81]
Kaneshiro (2009)	ARB	—	68	baPWV	Some concerns	[82]
Kim (2014)	ARB	NCT00496834	182	AIx, AIx@75, cfPWV	Some concerns	[83]
Lim (2026)	ARB	NCT05328310	98	AIx, AIx@75	Some concerns	[84]
Lim (2011)	ARB	—	60	cfPWV	Some concerns	[85]
Park (2014)	ARB	NCT01071122	132	AIx@75	Some concerns	[86]
Radchenko (2013)	ARB	—	59	AIx, cfPWV	High	[87]
Rehman (2007)	ARB	—	39	cfPWV	Some concerns	[88]
Rogers (2022)	ARB	—	141	AIx@75, cfPWV	High	[89]
Schneider (2008)	ARB	—	156	AIx	Some concerns	[90]
Shin (2019)	ARB	—	142	cfPWV	High	[91]
Spanos (2012)	ARB	—	29	AIx@75, cfPWV	High	[92]
Sun (2016)	ARB	—	65	cfPWV	Some concerns	[93]
Takami (2011)	ARB	—	50	AIx@75, baPWV	Some concerns	[94]
Tomiyama (2011)	ARB	—	113	baPWV	High	[95]
Xu (2019)	ARB	NCT01167153	118	baPWV	Some concerns	[96]
Schmieder (2017)	ARNi	NCT01870739	106	AIx, AIx@75, cfPWV	Some concerns	[97]
Agnoletti (2013)	CCB	—	66	AIx, cfPWV	Some concerns	[98]
Beaussier (2015)	CCB	NCT00224549	134	cfPWV	Some concerns	[99]
Liu (2021)	CCB	NCT02940548	47	baPWV	High	[100]
Mizuno (2016)	CCB	UMIN000010163 (Univers	89	baPWV	Some concerns	[101]
Pathapati (2015)	CCB	—	60	AIx, cfPWV	High	[102]
Shi (2017)	CCB	NCT01541189	81	baPWV	High	[103]
Takenaka (2012)	CCB	UMIN-ID: 000003323	59	AIx	Some concerns	[104]
Nowak (2019)	MRA	NCT01853553	60	AIx, cfPWV	Low	[105]
Kolwelter (2021)	SGLT2i	NCT03128528	71	cfPWV	Some concerns	[106]

ACEi = angiotensin-converting enzyme inhibitor; ARB = angiotensin receptor blocker; ARNi = angiotensin receptor-neprilysin inhibitor; CCB = calcium channel blocker; MRA = mineralocorticoid receptor antagonist; SGLT2i = sodium-glucose co-transporter 2 inhibitor; RoB 2 = Cochrane risk-of-bias tool for randomised trials, overall judgement.

**Table 4:** All pooled estimates. Effect measures are mean differences except for Objective 1b, which is a correlation pooled after Fisher z transformation and back-transformed. k = number of independent studies; N = total participants contributing.

Objective	Index	k	N	Estimate (95% CI)	*p*	I^2^ (%)	τ^2^	95% Prediction Interval
1a	Carotid–femoral PWV (m/s)	16	3095	+1.72 (+0.73 to +2.70)	0.002	96	2.947	−2.10 to +5.53
1a	Augmentation index (%)	10	1415	+4.51 (+1.36 to +7.65)	0.010	76	12.035	−4.11 to +13.13
1a	Brachial–ankle PWV (m/s)	7	1526	+2.24 (+1.38 to +3.10)	0.0007	56	0.438	+0.31 to +4.16
1a	Cardio-ankle vascular index	5	724	+1.00 (+0.29 to +1.71)	0.017	76	0.214	−0.68 to +2.68
1a	Augmentation index @75 bpm (%)	3	406	+2.25 (−1.10 to +5.59)	0.102	0	0.000	not estimable
1a	Effective arterial elastance (mmHg/mL)	7	899	+0.19 (−0.16 to +0.53)	0.237	90	0.066	−0.57 to +0.94
1a	Ea indexed to body surface area	3	669	+0.12 (−0.31 to +0.56)	0.340	52	0.020	not estimable
1a	Ventriculo-arterial coupling (Ea/Ees)	5	626	+0.06 (−0.32 to +0.43)	0.699	98	0.029	−0.64 to +0.75
1b	Stiffness–longitudinal strain (r)	4	—	−0.28 (−0.65 to +0.20)	0.162	79	—	not estimable
2	Augmentation index @75 bpm (%)	11	1432	−2.18 (−3.52 to −0.85)	0.004	73	1.359	−5.15 to +0.78
2	Carotid–femoral PWV (m/s)	18	1830	−0.11 (−0.40 to +0.18)	0.428	98	0.263	−1.23 to +1.01
2	Augmentation index (%)	13	1435	−1.32 (−6.48 to +3.83)	0.586	100	69.277	−20.37 to +17.72
2	Brachial–ankle PWV (m/s)	10	979	−0.56 (−1.40 to +0.28)	0.165	91	0.824	−2.82 to +1.70
3	Reflected-wave transit time (ms)	4	448	−5.09 (−11.45 to +1.27)	0.086	0	0.000	not estimable

All models are random effects with DerSimonian–Laird estimations of τ^2^ and Hartung–Knapp–Sidik–Jonkman confidence intervals. Positive values for Objective 1a indicate greater stiffness in HFpEF; negative values for Objective 2 favour the pre-specified drug class. Abbreviations are similar to those in Table 2.

**Table 5:** Pre-specified sensitivity analyses. Estimates are mean differences in the units of the index (m/s for pulse wave velocity, % for augmentation index, mmHg/mL for effective arterial elastance).

Objective	Index	Analysis	k	Estimate (95% CI)	*p*	I^2^ (%)
1a	Carotid–femoral PWV	Primary	16	+1.72 (+0.73 to +2.70)	0.002	96
1a	Carotid–femoral PWV	Restricted to clinically or haemodynamically defined HFpEF	10	+1.83 (+0.004 to +3.66)	0.050	95
1a	Augmentation index	Primary	10	+4.51 (+1.36 to +7.65)	0.010	76
1a	Augmentation index	Excluding SD/SE-ambiguous studies	6	+3.08 (−0.87 to +7.03)	0.102	—
1a	Augmentation index	Restricted to clinically or haemodynamically defined HFpEF	7	+3.16 (−0.38 to +6.70)	0.071	43
1a	Brachial–ankle PWV	Primary	7	+2.24 (+1.38 to +3.10)	0.0007	56
1a	Brachial–ankle PWV	Restricted to clinically or haemodynamically defined HFpEF	3	+2.10 (−1.73 to +5.94)	0.142	83
2	Augmentation index @75 bpm	Primary	11	−2.18 (−3.52 to −0.85)	0.004	73
2	Augmentation index @75 bpm	Excluding trials at high risk of bias	9	−2.28 (−3.79 to −0.76)	0.008	77
2	Augmentation index @75 bpm	Adding the four generic-inverse-variance trials reporting AIx@75	15	−1.36 (−2.78 to +0.07)	0.060	86
2	Carotid–femoral PWV	Primary	18	−0.11 (−0.40 to +0.18)	0.428	98
2	Carotid–femoral PWV	Excluding trials at high risk of bias	13	−0.21 (−0.41 to −0.02)	0.036	64
2	Carotid–femoral PWV	Adding the seven generic-inverse-variance trials reporting cfPWV	25	−0.09 (−0.36 to +0.18)	0.506	97
2	Augmentation index	Excluding trials at high risk of bias	9	−3.08 (−7.14 to +0.98)	0.118	94
2	Brachial–ankle PWV	Excluding trials at high risk of bias	6	−0.55 (−1.43 to +0.34)	0.172	84

k = number of contributing studies; SD = standard deviation; SE = standard error. Rows in which a restriction left fewer than three studies are omitted.

**Table 6:** Status of the four pre-specified predictions.

Prediction	Status	Basis
Every arterial stiffness domain is higher in HFpEF	Partially supported	Five indices significant in the primary analysis; prediction intervals include the null for all but brachial–ankle PWV; augmentation index loses significance and carotid–femoral PWV is only marginal (*p* = 0.050) when the analysis is restricted to clinically or haemodynamically defined HFpEF
Arterial stiffness tracks longitudinal dysfunction	Not supported	r = −0.28 (95% CI −0.65 to +0.20), k = 4, *p* = 0.162, I^2^ = 79%; two further studies were excluded because the second variable was not a measure of longitudinal function
Pharmacotherapy restores arterial phase relationships	Not supported as phase restoration	AIx@75 significant in the primary analysis but *p* = 0.060 with ten additional eligible trials; all contributing trials were conducted in hypertension; no timing index changed
Timing indices discriminate more sharply than scalar indices	Not testable	Only one timing index reached the pooling threshold: reflected-wave transit time, k = 4, −5.09 ms (95% CI −11.45 to +1.27), *p* = 0.086

**Table 7:** Pre-specified drug classes mapped onto the phasic framework, with the evidence actually available in this synthesis. “Not estimable” indicates that fewer than three eligible trials of that class reported the index, so no class-specific pooled estimate could be computed.

Drug Class	Effect on PWV	Effect on AIx	Phasic Target	Eligible RCTs Here
ACEi/ARB	No pooled effect	AIx@75 reduced	Arterial diastole (wave reflection)	≈two-thirds of eligible trials
ARNi	Not estimable	Not estimable separately	Arterial diastole + anti-fibrotic	few trials, none pooled by class
SGLT2 inhibitors	Not estimable	Not estimable	Arterial diastole (endothelial, anti-fibrotic)	almost all trials in diabetes, excluded
Calcium channel blockers	No pooled effect	No pooled effect	Arterial diastole (reflection site tone)	several trials, mostly head-to-head
Organic nitrates	Not estimable	Not estimable	Arterial diastole (reflection amplitude)	isolated trials only
MRA	Not estimable	Not estimable	Arterial systole (anti-fibrotic, wall recoil)	isolated trials only

AIx = augmentation index; AIx@75 = augmentation index corrected to 75 beats per minute; PWV = pulse wave velocity; RCT = randomised controlled trial. Other abbreviations are similar to those in Table 2.

**Table 8:** GRADE certainty of evidence for the principal pooled outcomes.

Outcome	Risk of Bias	Inconsistency	Indirectness	Imprecision	Publication Bias	Certainty
1a—Carotid–femoral PWV	Serious	Serious (I^2^ = 96%; prediction interval includes the null)	Not serious	Not serious (k = 16)	Undetected (Egger +1.18, *p* = 0.599)	Low
1a—Augmentation index	Serious	Serious (I^2^ = 76%; prediction interval includes the null)	Not serious	Serious (wide interval)	Undetected (Egger +0.52, *p* = 0.684)	Low
1a—Brachial–ankle PWV	Serious	Not serious (I^2^ = 56%; prediction interval excludes the null)	Not serious	Serious (k = 7)	Not assessed (k < 10)	Low
2—AIx@75	Serious (12 of 36 trials at high risk)	Serious (I^2^ = 73%)	Serious—all contributing trials in hypertension, none in HFpEF	Serious (does not survive ten further trials)	Undetected (Egger +0.66, *p* = 0.350)	Low
2—Carotid–femoral PWV	Serious	Very serious (I^2^ = 98%; significance changes when high-risk trials are excluded)	Serious	Not serious (k = 18)	Undetected (Egger +0.87, *p* = 0.672)	Very low
2—Brachial–ankle PWV	Serious	Serious (I^2^ = 91%)	Serious	Serious	Suspected (Egger −3.64, *p* = 0.030)	Very low
3—Reflected-wave transit time	Serious	Not serious (I^2^ = 0%)	Not serious	Very serious (k = 4)	Not assessed (k < 10)	Very low
1b—Stiffness and longitudinal strain	Serious	Serious (I^2^ = 79%)	Serious	Very serious (k = 4)	Not assessed (k < 10)	Very low

## Data Availability

All extracted data, analysis code, and files necessary to reproduce the full results are provided in the Supplementary Materials. The review protocol is deposited as a time-stamped registration on the Open Science Framework (https://doi.org/10.17605/OSF.IO/FT6Q4). The database contains, for each effect, the exact source of the value—including the text, table, Supplementary Material, or figure—so that any reader can reproduce the extraction.

## Supplementary Materials

The following supporting information can be downloaded at https://www.mdpi.com/article/10.3390/diagnostics16213599/s1. Table S1: PRISMA 2020 checklist; Table S2: PRISMA-S checklist for reporting literature searches; Table S3: Full search strategies for MEDLINE/PubMed, Scopus and Web of Science, with the date each was run and the number of records returned; Table S4: The four analysed datasets (Objectives 1a, 1b, 2, and 3) as used, with one row per contrast; Table S5: Overlapping publications, with the trial registration or cohort on which the overlap was detected, and the report retained for each pooled estimate; Table S6: Each unit harmonisation and dispersion conversion applied, with its arithmetic; Table S7: The 140 full texts excluded at the full-text stage, each with its exclusion code and reason; Table S8: The 236 studies included in the qualitative synthesis, with their references, designs, populations, and indices reported; Table S9: Newcastle–Ottawa Scale assessments for the observational studies by domain; Table S10: Cochrane RoB 2 assessments for the randomised trials by domain; Table S11: Leave-one-out analyses for each pooled estimate with four or more contributing studies; Table S12: The 28 eligible studies reporting at least one phasic index, with the variable measured, its unit, the population, the comparator, and the reason each did or did not contribute; Table S13: Eligible studies that did not contribute to any pooled estimate, each with its recorded reason; Table S14: Comparisons in which both randomised arms contained a pre-specified drug class; Table S15: Populations of the randomised trials contributing to Objective 2; Table S16: Full texts recovered after peer review, with its inclusion decision and reason for each; Table S17: Register of corrections declared in this revision, each with the estimates before and after; Table S18: Verification record, with each value entering the pooled estimate and the second author’s recorded responses.

## Abbreviations

The following abbreviations are used in this manuscript:

ACEi	Angiotensin-converting enzyme inhibitor
AIx	Augmentation index
AIx@75	Augmentation index, corrected to a heart rate of 75 beats per minute
ARB	Angiotensin receptor blocker
ARNi	Angiotensin receptor-neprilysin inhibitor
baPWV	Brachial–ankle pulse wave velocity
CAVI	Cardio-ankle vascular index
CCB	Calcium channel blocker
cfPWV	Carotid–femoral pulse wave velocity
CI	Confidence interval
Ea	Effective arterial elastance
Ees	End-systolic elastance
EF	Ejection fraction
GLS	Global longitudinal strain
HFpEF	Heart failure with preserved ejection fraction
HKSJ	Hartung–Knapp–Sidik–Jonkman
LV	Left ventricular
MD	Mean difference
MRA	Mineralocorticoid receptor antagonist
NOS	Newcastle–Ottawa Scale
OSF	Open Science Framework
PRISMA	Preferred Reporting Items for Systematic Reviews and Meta-Analyses
PWV	Pulse wave velocity
RoB 2	Cochrane risk-of-bias tool for randomised trials, version 2
SD	Standard deviation
SE	Standard error
SGLT2i	Sodium-glucose co-transporter 2 inhibitor
