# Supplementary material for: Phasic Arterial Mechanics and Ventriculo-Arterial Dyssynchrony in Heart Failure with Preserved Ejection Fraction: A Systematic Review and Meta-Analysis of Arterial Systole, Arterial Diastole and Pharmacological Phase Restoration
Source: Diagnostics (Basel). 2026 Nov 1;16(21):3599. doi: 10.3390/diagnostics16213599 (PMC13761822; doi:10.3390/diagnostics16213599)
Supplement: Supplementary file 1 [file diagnostics-16-03599-s001.zip › Supplementary_Tables_S1_S2_PRISMA_checklists.pdf]

Supplementary Materials

Phasic Arterial Mechanics and Ventriculo-Arterial Dyssynchrony in Heart Failure with Preserved Ejection Fraction: A Systematic Review and Meta-Analysis of Arterial Systole, Arterial Diastole and Pharmacological Phase Restoration

Georgică Târtea, Cristina Văcărescu, Dragoș Cozma

Protocol registered on the Open Science Framework: https://doi.org/10.17605/OSF.IO/FT6Q4

This file contains Table S1 (PRISMA 2020 checklist) and Table S2 (PRISMA-S checklist). Tables S3 to S18 are supplied as a separate workbook, Supplementary_Tables.xlsx, and Figure S1 as a separate image file.

Table S1. PRISMA 2020 checklist

Location is given as the section of the manuscript in which the item is addressed. "Protocol" refers to the registered protocol deposited on the Open Science Framework, which is cited in Section 2.1.

#	Item	Checklist item	Location where the item is reported
**TITLE**			
1	Title	Identify the report as a systematic review.	Title ("A Systematic Review and Meta-Analysis"); article type declared as Systematic Review
**ABSTRACT**			
2	Abstract	See the PRISMA 2020 for Abstracts checklist.	Abstract, structured as Background/Objectives, Methods, Results, Conclusions
**INTRODUCTION**			
3	Rationale	Describe the rationale for the review in the context of existing knowledge.	Section 1, paragraphs 1–4
4	Objectives	Provide an explicit statement of the objective(s) or question(s) the review addresses.	Section 1, final paragraph (four stated predictions); Section 2.2 (PECO per objective)
**METHODS**			
5	Eligibility criteria	Specify the inclusion and exclusion criteria and how studies were grouped for the syntheses.	Section 2.2; exclusion codes E1–E11 listed in Section 2.2 and defined in Table S5
6	Information sources	Specify all databases, registers, websites, organisations, reference lists and other sources searched, and the date each was last searched.	Section 2.3 (searches run on 10 September 2026); Table S3
7	Search strategy	Present the full search strategies for all databases, registers and websites, including any filters and limits used.	Table S3 (all 12 queries verbatim, across three databases)
8	Selection process	Specify the methods used to decide whether a study met the inclusion criteria, including how many reviewers screened each record and whether they worked independently.	Section 2.4; screening rubric deposited with the protocol
9	Data collection process	Specify the methods used to collect data from reports, including how many reviewers collected data and whether they worked independently.	Sections 2.4 and 2.9, including the two binding extraction rules and the independent verification of every pooled value; extraction schema deposited with the protocol
10a	Data items — outcomes	List and define all outcomes for which data were sought.	Section 2.2 (accepted arterial and phasic indices)
10b	Data items — other variables	List and define all other variables for which data were sought.	Section 2.4
11	Study risk of bias assessment	Specify the methods used to assess risk of bias, including details of the tool(s) used and how many reviewers assessed each study.	Section 2.5; Tables S9 and S10
12	Effect measures	Specify for each outcome the effect measure used in the synthesis or presentation of results.	Section 2.7; Table 4
13a	Synthesis —	Describe the processes used to decide which studies were eligible for each	Section 2.7 (pooling threshold k ≥ 3; index families never pooled together); Section 2.6

	eligibility	synthesis.	(overlap)
13b	Synthesis — data preparation	Describe any methods required to prepare the data for presentation or synthesis.	Section 2.7 (unit harmonisation and dispersion conversions); Table S6
13c	Synthesis — tabulation	Describe any methods used to tabulate or visually display results of individual studies and syntheses.	Tables 1–3; Figures 3–5
13d	Synthesis — methods	Describe any methods used to synthesise results and provide a rationale for the choice(s). Identify the model(s), method(s) to identify heterogeneity, and software package(s) used.	Section 2.7 (random effects, DerSimonian–Laird τ^2 with Hartung–Knapp–Sidik–Jonkman intervals; τ^2, I^2, Q, 95% prediction intervals); analysis code deposited
13e	Synthesis — subgroups	Describe any methods used to explore possible causes of heterogeneity among study results.	Section 2.7 (subgroups by drug class and by design; leave-one-out at $k \geq 4$); Table S11
13f	Synthesis — sensitivity	Describe any sensitivity analyses conducted to assess robustness of the synthesised results.	Section 2.7 (four pre-specified analyses); Section 3.8; Table 5
14	Reporting bias assessment	Describe any methods used to assess risk of bias due to missing results in a synthesis.	Section 2.7 (funnel plots and Egger's test at $k \geq 10$); Section 3.7; Figure 5
15	Certainty assessment	Describe any methods used to assess certainty (or confidence) in the body of evidence for an outcome.	Section 2.7 and Section 4.7; GRADE domains assessed for every principal pooled outcome in Table 8
**RESULTS**			
16a	Study selection — flow	Describe the results of the search and selection process, from the number of records identified through to the number of studies included, ideally using a flow diagram.	Section 3.1; Figure 2
16b	Study selection — exclusions	Cite studies that might appear to meet the inclusion criteria but were excluded, and explain why.	Table S7 (all 140 full texts excluded, each with code and reason)
17	Study characteristics	Cite each included study and present its characteristics.	Tables 1 and 2 (studies contributing to synthesis); Table S8 (all 236 included studies)
18	Risk of bias in studies	Present assessments of risk of bias for each included study.	Table 1 (NOS column) and Table 2 (RoB 2 column); Tables S9 and S10 give every domain
19	Results of individual studies	For all outcomes, present for each study summary statistics for each group and an effect estimate with its precision.	Figures 3 and 4 (forest plots); Tables S4a–S4d (every contrast as used)
20a	Results of syntheses — studies	For each synthesis, briefly summarise the characteristics and risk of bias among contributing studies.	Sections 3.2, 3.3, 3.5, 3.7
20b	Results of syntheses — estimates	Present results of all statistical syntheses, including summary estimates and their precision and measures of heterogeneity.	Table 4 (all pooled estimates with 95% CI, p, I^2, τ^2 and prediction interval); Sections 3.3–3.6
20c	Results of syntheses — heterogeneity	Present results of all investigations of possible causes of heterogeneity among study results.	Section 3.3 (sources of heterogeneity discussed by study); Section 3.5 (subgroups by class); Table S11
20d	Results of syntheses — sensitivity	Present results of all sensitivity analyses conducted to assess robustness of the synthesised results.	Section 3.8; Table 5
21	Reporting biases	Present assessments of risk of bias due to missing results for each synthesis assessed.	Section 3.7; Figure 5
22	Certainty of evidence	Present assessments of certainty (or confidence) in the body of evidence for each outcome assessed.	Section 4.7
**DISCUSSION**			
23a	Discussion — interpretation	Provide a general interpretation of the results in the context of other evidence.	Sections 4.1–4.4
23b	Discussion — limitations of evidence	Discuss any limitations of the evidence included in the review.	Section 4.7
23c	Discussion — limitations of	Discuss any limitations of the review processes used.	Section 4.7 and Sections 2.8 and 2.9

	review		
23d	Discussion — implications	Discuss implications of the results for practice, policy and future research.	Sections 4.2, 4.4 and 5
**OTHER INFORMATION**			
24a	Registration	Provide registration information for the review, including the register name and registration number, or state that the review was not registered.	Section 2.1 (Open Science Framework, https://doi.org/10.17605/OSF.IO/FT6Q4; registration retrospective with respect to data collection, declared in the protocol)
24b	Protocol	Indicate where the review protocol can be accessed, or state that a protocol was not prepared.	Section 2.1; Data Availability Statement
24c	Amendments	Describe and explain any amendments to information provided at registration or in the protocol.	Section 2.8 (four protocol decisions, each declared)
25	Support	Describe sources of financial or non-financial support and the role of the funders.	Funding statement (none)
26	Competing interests	Declare any competing interests of review authors.	Conflicts of Interest statement
27	Availability of data, code and other materials	Report which of the following are publicly available and where: template data collection forms; data extracted; data used in all analyses; analytical code; any other materials.	Data Availability Statement; Tables S1–S18; the Open Science Framework project (https://doi.org/10.17605/OSF.IO/FT6Q4) holds the extraction database, the analysis code and the Stata do-files

Table S2. PRISMA-S checklist for reporting literature searches

PRISMA-S is the search-reporting extension of PRISMA 2020 (Rethlefsen et al., Syst Rev 2021;10:39).

#	Item	Checklist item	Location / response
**INFORMATION SOURCES AND METHODS**			
1	Database name	Name each individual database searched, stating the platform for each.	Section 2.3 and Table S3: MEDLINE via PubMed; Scopus (Elsevier); Web of Science Core Collection (Clarivate)
2	Multi-database searching	If databases were searched simultaneously on a single platform, state the name of the platform, listing all databases searched.	Not applicable — each database was searched separately on its own platform
3	Study registries	List any study registries searched.	None searched. Trial registration numbers were extracted from the included reports and used to detect overlap (Section 2.6)
4	Online resources and browsing	Describe any online or print source purposefully searched or browsed.	Section 2.3: reference lists of all included articles, and of McDonald's Blood Flow in Arteries, 7th edition, were hand-searched
5	Citation searching	Indicate whether cited references or citing references were examined.	Backward citation searching only (reference lists), as above. No forward citation searching
6	Contacts	Indicate whether additional studies or data were sought by contacting authors.	No authors were contacted. Values not printed in the text or tables were left missing rather than requested; this is stated in Section 2.4 and is a limitation noted in Section 4.7
7	Other methods	Describe any additional information sources or search methods used.	None
**SEARCH STRATEGIES**			
8	Full search strategies	Include the search strategies for each database and information source, copied and pasted exactly as run.	Table S3 — all 12 queries, verbatim
9	Limits and restrictions	Specify that no limits were used, or describe any limits or restrictions applied and provide justification.	Section 2.3: no language restriction and no date restriction. The only filter applied was an animal-study exclusion, embedded in each query and visible in Table S1
10	Search filters	Indicate whether published search filters were used and, if so, describe the filter and cite it.	An adaptation of the standard PubMed animal-exclusion clause NOT ("animals"[mh] NOT "humans"[mh]) and, for Objective 2, the Cochrane Highly Sensitive Search Strategy for randomised trials. Both appear in full in Table S1
11	Prior work	Indicate when search strategies from other reviews were adapted or reused.	No strategy was adapted from another review
12	Updates	Report the methods used to update the search(es).	The search was run once, in 10 September 2026, and was not updated before submission
13	Dates of searches	For each search strategy, provide the date when the last search occurred.	10 September 2026, for all three databases (Table S1)
**PEER REVIEW**			
14	Peer review	Describe any search peer review process.	The strategies were not formally peer reviewed by an information specialist. They were revised once after a pilot run showed the animal-exclusion clause had not been applied to the exported sets, and the corrected queries — the ones reported in Table S1 — are those whose results were used. This is stated as a limitation in Section 4.7
**MANAGING RECORDS**			
15	Total records	Document the total number of records identified from each database and other information sources.	Section 3.1 and Figure 2: MEDLINE/PubMed 1 072; Scopus 1 665; Web of Science 1 098; total 3 835

16	Deduplication	Describe the processes and any software used to deduplicate records from multiple sources.	Section 2.4: staged deduplication on PMID, then DOI, then normalised title and year, within and across databases; 1 792 duplicates removed. The deduplication code is deposited with the analysis code
